# Epidemic Dynamics and Intervention Measures in Campus Settings Based on Multilayer Temporal Networks

**DOI:** 10.3390/e27050543

**Published:** 2025-05-21

**Authors:** Xianyang Zhang, Ming Tang

**Affiliations:** 1 School of Physics and Electronic Science, East China Normal University, Shanghai 200241, China; 51254700007@stu.ecnu.edu.cn; 2 Shanghai Key Laboratory of Multidimensional Information Processing, East China Normal University, Shanghai 200241, China

**Keywords:** multilayer temporal network, campus epidemics, intervention strategies

## Abstract

This study simulates the spread of epidemics on university campuses using a multilayer temporal network model combined with the SEIR (Susceptible–Exposed–Infectious–Recovered) transmission model. The proposed approach explicitly captures the time-varying contact patterns across four distinct layers (Rest, Dining, Activity, and Academic) to reflect realistic student mobility driven by class schedules and spatial constraints. It evaluates the impact of various intervention measures on epidemic spreading, including subnetwork closure and zoned management. Our analysis reveals that the Academic and Activity layers emerge as high-risk transmission hubs due to their dynamic, high-density contact structures. Intervention measures exhibit layer-dependent efficacy: zoned management is highly effective in high-contact subnetworks, its impact on low-contact subnetworks remains limited. Consequently, intervention measures must be dynamically adjusted based on the characteristics of each subnetwork and the epidemic situations, with higher participation rates enhancing the effectiveness of these measures. This work advances methodological innovation in temporal network epidemiology by bridging structural dynamics with SEIR processes, offering actionable insights for campus-level pandemic preparedness. The findings underscore the necessity of layer-aware policies to optimize resource allocation in complex, time-dependent contact systems.

## 1. Introduction

The spread of epidemics has consistently been a central issue in the public health domain. Through the construction of mathematical models [1,2], researchers can simulate and predict the process of disease diffusion [3,4] and provide scientific evidence for the formulation of epidemic prevention and control strategies [5,6]. In recent years, complex network theory has become widely applied in this research field. Complex network models abstract social relationships and contact behaviors into nodes and edges, enabling detailed descriptions of epidemic transmission pathways [7,8] and helping researchers reveal the impact of network structure on the spatiotemporal patterns of disease spreading [9].

The multilayer network model enhances the application of complex network models by incorporating interactions between different groups or settings, such as households, schools, and workplaces [10]. For instance, Liu et al. developed an epidemic model that incorporates different reactive class closure strategies for schools, households, and workplaces. The results indicated that class closure measures, when implemented at the appropriate time, can significantly suppress the outbreak of epidemics [11]. Moreover, Zeng et al. constructed a three-layer transportation network that integrates various commuting modes and commuting-related infections to forecast the epidemic situation in Shanghai during the 2022 outbreak. Their findings revealed that regional population size significantly impacts the total number of infection cases [12]. Chen et al. proposed a multilayer network model that integrates a structurally fixed social contact network with a time-varying commuting network. They found a strong correlation between the coverage and duration of non-pharmaceutical interventions (NPIs) and the mitigation of pandemics [13]. Chen et al. developed an epidemic spreading model on a dual-layer network with non-Markovian interlayer travel infections. Their results showed that when travel time exceeds a critical threshold, the epidemic threshold increases significantly, and a dual-threshold phenomenon occurs [14].

Previous studies have primarily focused on large-scale spatial networks at the city or national level [15]. However, high-density closed systems such as campuses have not been extensively studied. The high population density and frequent interpersonal interactions within closed spaces can accelerate the spread of epidemics, potentially leading to severe public health crises [16]. Therefore, investigating disease transmission in closed, densely populated environments is of significant importance [17]. Hekmati et al. constructed a closed-space air transmission model that comprehensively considers factors such as room ventilation rates, mask usage efficiency, and occupant infection rates, emphasizing that air transmission is the primary route for the spread of COVID-19 [18]. Additionally, Tatapudi used an individual-based model to explore changes in epidemic spreading at U.S. universities under conditions of partial or full reopening [19]. Such individual-based models are often used in studies of management measures, such as in-person teaching models, contact tracing, and non-pharmaceutical interventions (NPIs) like vaccination [20,21]. In previous campus network studies, the transitions of nodes across different social layers (locations) have often been neglected, with social interactions across different places aggregated through a single-layer network. However, in many real-world scenarios, this assumption fails to effectively describe the interactions and influences between different groups or settings.

Multilayer temporal networks provide a more accurate representation of complex social interaction dynamics compared to single-layer temporal networks, particularly in capturing the heterogeneous nature of real-world contact patterns [22]. In campus environments, distinct social layers exhibit unique interaction patterns with varying temporal characteristics [23]. The multilayer framework enables more precise modeling of these temporal variations, as demonstrated by [24], who established fundamental theoretical results for epidemic thresholds in such networks. Recent work by [25] further developed analytical tools for characterizing spreading processes across interconnected temporal layers, while [26] provided comprehensive mathematical foundations for these models. This modeling approach allows researchers to systematically evaluate prevention measures through scenario-based analysis of interaction behaviors and spreading patterns [27,28,29,30], offering significant advantages over traditional single-layer representations.

This paper proposes a campus epidemic spreading modeling framework based on multilayer temporal networks (MTNs), which overcomes the limitations of traditional single-layer or static network models. Combining the Susceptible–Exposed–Infectious–Recovered (SEIR) model, our model integrates the heterogeneity and time-varying interaction patterns across multiple social layers and captures the spatiotemporal dynamics of student mobility across different locations. This approach addresses the oversimplification of interlayer individual migration in existing studies. Compared to other single-layer multilayer or temporal models, this framework not only reveals the impact of interlayer temporal migration on epidemic transmission but also quantifies how layer-specific intervention efficacy varies with temporal network structures, revealing that high-contact layers require fundamentally different control strategies than low-contact layers. These innovations offer transformative decision support tools, enabling administrators to optimize resource allocation by targeting critical space–time transmission hotspots while minimizing operational disruptions.

## 2. Materials and Methods

### 2.1. Data

The Minhang campus of East China Normal University, as a typical high-density closed campus, has the following characteristics:

1. The total student population is approximately 21,800, with a dormitory occupancy rate exceeding 95%, and the dormitories are shared by 2 to 4 students.
2. Fixed class hours are scheduled daily (from 7:00 a.m. to 9:15 p.m.), and the course schedule drives the spatiotemporal distribution of the student population.
3. Functional areas are concentrated (e.g., classroom buildings, canteens, sports complexes), with frequent interarea movement.

These characteristics make it an ideal case for studying epidemic spreading in closed environments.

Course schedule data were sourced from the official 2023 fall semester timetable of East China Normal University, covering 2160 courses across 29 departments. Venue capacity data were obtained through on-site surveys. For example, classroom capacities range from 24 to 278 people, while the peak period crowd density in canteens is 2.5 people per square meter (with a sampling measurement standard deviation of ±0.3). Additionally, according to data from the East China Normal University Logistics Service Center, the four main canteens on the Minhang campus have a total of 10 floors and 5609 seats, with individual floor capacities ranging from 360 to 910 people.

This model divides the day into 18 discrete time steps, representing the period from 7:00 a.m. to 10:00 p.m. Each time step corresponds to 50 min, aligning with the interval between a class and its break in the schedule as shown in Table 1.

### 2.2. Multilayer Temporal Campus Network Model

**Figure 1:**
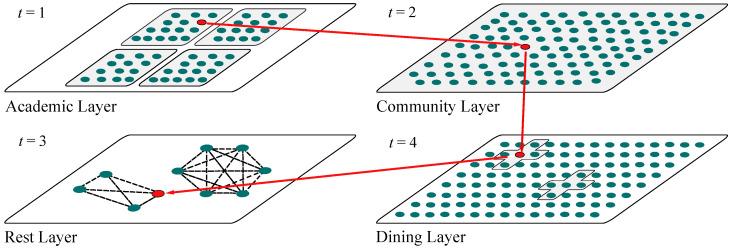
The schematic diagram of East China Normal University’s multilayer time-varying network shows active individuals (red nodes) migrating across four subnetworks (R/D/C/A layers) via red arrows from t=1 to t=4.

This study employs a multilayer temporal network framework to model the physical contact dynamics among students on the Minhang campus of East China Normal University. The nodes in the networks represent students in this agent-based modeling approach, while the edges within each layer represent the contact relationships between individuals in different locations. The model consists of four subnetworks—Rest Layer (R), Dining Layer (D), Community Layer (C), and Academic Layer (A)—with active nodes capable of migrating between layers (locations). Figure 1 illustrates the migration of active individuals between the four layers from time t=1 to t=4 in this multilayer temporal network model.

The day is divided into $T_{d}=18$ discrete time steps representing the period from 7:00 a.m. to 10:00 p.m., with each time step corresponding to 50 min, aligning with the interval between a class and its break, as shown in the timetable. During the remaining time, individuals are assumed to be in the Rest Layer, not participating in any other activities and without disease transmission.

The migration rules for active individuals between subnetworks are outlined as follows:

1. According to the official timetable, individuals prioritize entering the Academic Layer (A) based on the schedule of the day’s classes;
2. When individuals are not in the Academic Layer and the Dining Layer is open, they will enter the Dining Layer (D);
3. When individuals are not in the Academic Layer and the Dining Layer is closed, they will enter the Community Layer (C) with probability $P_{c}\left(t\right)$. This probability is given by $P_{c}\left(t\right)=t\sum_{i=1}^{t}A\left(i\right)/\sum_{\tau =1}^{18}\tau \sum_{i=1}^{\tau }A\left(i\right)$, where A(i) is an indicator function, which takes the value of 1 when the individual is in the Academic Layer at time *i* and 0 otherwise. The probability grows non-linearly with time and the accumulation of class duration, making the tendency to enter the Community Layer significantly stronger during the evening hours (when *t* is larger). To constrain the probability within the [0, 1] range, it is further normalized by the total cumulative amount $\sum_{\tau =1}^{18}\tau \sum_{i=1}^{\tau }A\left(i\right)$;
4. Individuals stay in each subnetwork for one time step, completing interactions within the layer before choosing the next active subnetwork.

Since the number of nodes an individual can contact within each layer is limited, the network structure at each time step can be modeled as a random network with a contact probability of $1/N_{P}\left(t\right)$, where $N_{P}\left(t\right)$ represents the number of people at a given location at time *t* (e.g., the dormitory in the Rest Layer or the classroom in the Academic Layer).

The model carefully simulates the actual social and demographic structure of a university campus, considering factors such as population age structure, dormitory distribution, class sizes, and canteen sizes. It is worth noting that faculty and staff are excluded from the model, as their daily work during the pandemic is presumed to involve effective disinfection, isolation, and other preventive measures, maintaining a low contact risk while keeping a large social distance from students.

Next, in the constructed multilayer temporal networks, the SEIR (Susceptible–Exposed–Infectious–Recovered) model is used to describe the disease transmission processes. Specifically, susceptible nodes (S) become infected after contacting infectious nodes (I) with transmission rate β. Once infected, nodes move to the exposed state (E), where they are not infectious. Exposed nodes transition to the infectious state (I) at a rate σ, and infectious nodes recover to the removed state (R) at rate γ.

To better illustrate the model’s construction and the parameters used, Table 2 summarizes the detailed information about network parameters and epidemic parameters. Network parameters include the number of departments, dormitory capacities, and total number of courses on campus. Epidemic parameters include values such as the basic reproduction number $R_{0}$, infection rate β, and recovery rate γ. These parameter values were derived from the existing literatures and campus-specific survey data, ensuring that the model accurately reflects the real disease transmission dynamics within the campus during epidemics.

## 3. Results

### 3.1. Epidemic Situation Analysis in Multilayer Coupled Networks and Their Subnetworks

The epidemic spreading processes at East China Normal University’s Minhang campus were simulated on a multilayer temporal network, where each time step corresponded to 50 min, and the total simulation duration represented one semester. Our analysis focused on short-term transmission dynamics where viral evolution and host immunity changes can be neglected [34], which are consistent with established outbreak response timelines for single-strain dominance periods. The Monte Carlo simulations took into account individual interactions and migration behaviors in different locations such as classrooms, dormitories, canteens, and public areas. We simulated the epidemic spreading under different basic reproduction numbers and evaluated the contribution of each subnetwork to epidemic prevention and control.

**Figure 2:**
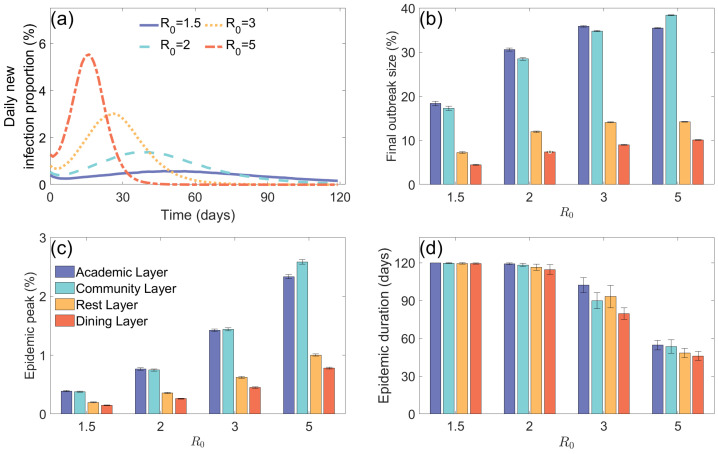
The infection situation in various subnetworks of the campus under different basic reproduction numbers. (**a**) Daily new infection proportion (%) over time for different basic reproduction numbers ($R_{0}=1.5,2,3,5$). The blue solid line, cyan dashed line, yellow dotted line, and red dotted–dashed line represent $R_{0}=1.5,2,3,5$, respectively. (**b**) Final outbreak size (%) for each layer (Academic, Community, Rest, and Dining) under varying $R_{0}$ values. Blue, cyan, yellow, and red represent the Academic, Community, Rest, and Dining layers, respectively. Error bars represent 95% confidence intervals. (**c**) Epidemic peak (%) for each layer at different $R_{0}$ values. (**d**) Epidemic duration (days) for each layer across different $R_{0}$ values.

Figure 2a shows the dynamic changes in the daily new infection proportion under different basic reproduction numbers ($R_{0}=1.5,2,3,5$). The results demonstrate that as $R_{0}$ increases, both the final outbreak size and epidemic peak increase significantly, with the epidemic peak occurring earlier. Based on this, Figure 2b–d further analyze the outbreak characteristics of each subnetwork. Figure 2b,c show that the the final outbreak size and epidemic peak in the Rest Layer are always the lowest; the Academic and Community Layers have significantly higher the final outbreak size and epidemic peak due to longer individual stay times and broader contact ranges; the Dining Layer shows relatively low the final outbreak size and epidemic peak, mainly because the contact time between individuals is shorter. Figure 2d indicates that the epidemic duration shortens as $R_{0}$ increases, with the Rest Layer having the shortest epidemic duration due to its smaller contact range and longer stay time.

### 3.2. The Suppressive Effects of Subnetwork (Location) Closure Measures

We further adjusted the distribution of individuals in different subnetworks by closing specific layers, thereby assessing their impacts on the epidemic situations. The main measures considered are defined as follows:

1. Closing the Community Layer: Nodes and edges originally belonging to the Community Layer at time *t* were entirely redistributed to the Academic Layer (C→A) or to the Rest Layer (C→R);
2. Closing the Academic Layer: Nodes and edges originally belonging to the Academic Layer at time *t* were entirely redistributed to the Community Layer (A→C) or to the Rest Layer (A→R).

**Figure 3:**
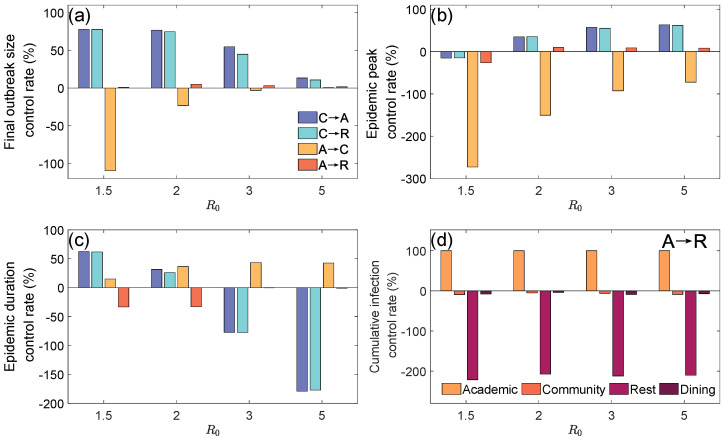
The suppressive effects of four subnetwork closure measures on the epidemic situation under different basic reproduction numbers. (**a**) Final outbreak size control rate (%) for each intervention measure across varying $R_{0}$ values. Blue, cyan, yellow, and red represent C→A (Community to Academic), C→R (Community to Rest), A→C (Academic to Community), and A→R (Academic to Rest), respectively. (**b**) Epidemic peak control rate (%) for each intervention measure at different $R_{0}$ values. (**c**) Epidemic duration control rate (%) for each intervention measure across varying $R_{0}$ values. (**d**) Cumulative infection control rate (%) for each layer under the A→R intervention measure across different $R_{0}$ values. Warm orange, bright red, deep purple-red, and deep burgundy represent Academic, Community, Rest, and Dining, respectively.

Figure 3a–c show the changes in the final outbreak size control rate, epidemic peak control rate, and epidemic duration control rate under different basic reproduction numbers and subnetwork closure measures. The control rate indicator is calculated as

(1) $$\Delta I=\frac{I_{o}-I_{i}}{I_{o}},$$

where $I_{o}$ and $I_{i}$ represent the values of the indicator without and with control measures, respectively.

Figure 3a shows that when $R_{0}=1.5$, the strategy of closing the Community Layer achieved a final outbreak size control rate of approximately 78%, which is quite effective. In contrast, the strategy of closing the Academic Layer and migrating to the Community Layer had a control rate of −110%, while migrating to the Rest Layer had almost no effect. This is likely because the contact probability in the model is inversely proportional to the number of people in a location, causing the effective contact rate in the Academic and Rest Layers to remain equivalent and within-layer contact to be homogeneous (e.g., fixed groups in classrooms or dormitories). After migration, the overall infection risk did not change significantly. On the other hand, the Community Layer, due to its heterogeneous contact pattern (e.g., random mixing between groups), created an independent transmission acceleration path. Closing this layer cut off such high-risk contacts completely, significantly reducing the final outbreak size. As $R_{0}$ increased, the final outbreak size control rate for all strategies tended to zero. When $R_{0}=5$, the effects of the strategies on the infection proportion were not significant, as the increased viral transmission ability at high $R_{0}$ made local interventions less effective at curbing the overall spreading.

Figure 3b further indicates that, at $R_{0}=1.5$, the epidemic peak control rate for all subnetwork closure strategies is negative, suggesting that under lower $R_{0}$, subnetwork closure strategies not only fail to effectively suppress the epidemic peak, but also have a negative effect. As $R_{0}$ increases, the strategy of closing the Community Layer significantly improves the epidemic peak control rate.

From Figure 3c, it can be seen that when $R_{0}=1.5$, the strategy of closing the Community Layer resulted in an epidemic duration control rate close to 70%, effectively controlling the epidemic’s development. However, as $R_{0}$ increased, the enhanced viral transmission ability and sustained infections led to a prolonged epidemic duration. Although the strategy of closing the Academic Layer and migrating to the Community Layer maintained the duration control rate at 50%, the epidemic spread more rapidly, significantly reducing the effectiveness of control measures.

Figure 3d clearly shows the impact of the strategy of closing the Academic Layer and migrating to the Rest Layer on the final outbreak size in each layer. From the figure, it is evident that the final outbreak size control rate in the Rest Layer significantly decreases. This is due to a large influx of population, which significantly encreases the population density in the Rest Layer, thereby raising the contact frequency and viral infection risk within that layer, which to some extent promotes the spread of the epidemic [as seen in Figure 3a]. This also causes the control rates in the Community Layer and Dining Layer to be slightly greater than zero.

**Figure 4:**
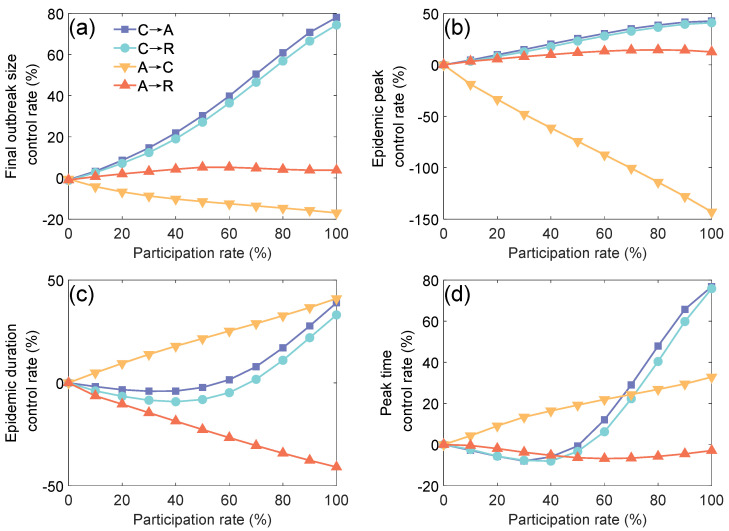
The impact of participation rate on epidemic control measures for four subnetwork closure measures. (**a**) Final outbreak size control rate (%) for each intervention measure under varying participation rates. Blue, cyan, yellow, and red represent C→A (Community to Academic), C→R (Community to Rest), A→C (Academic to Community), and A→R (Academic to Rest), respectively. (**b**) Epidemic peak control rate (%) for each intervention measure under varying participation rates. (**c**) Epidemic duration control rate (%) for each intervention measure under varying participation rates. (**d**) Peak time control rate (%) for each intervention measure under varying participation rates.

Next, we explored the impact of the actual participation rate (the proportion of individuals involved) under different control strategies on epidemic control. Figure 4 shows the effects of four subnetwork closure strategies on epidemic indicators at a basic reproduction number $R_{0}=2$ under varying participation rates.

Figure 4a,b show that the final outbreak size control rate and epidemic peak control rate follow a similar trend as the participation rate changes. When the Community Layer is closed, both final outbreak size infection control rate and the epidemic peak control rate increase significantly with the participation rate, suggesting that increasing the participation rate for this strategy can effectively curb epidemic spreading. However, under the strategy of closing the Academic Layer, an increase in the participation rate leads to a slight decrease in the control rate of migration to the Community Layer. This phenomenon occurs because, after the migration from the Academic Layer to the Community Layer, the Community Layer, as a high-contact area, experiences an increase in population density and contact frequency, which in turn exacerbates the risk of virus transmission.

Figure 4c,d show the differences in the impact of the participation rate on epidemic duration. In the strategy of closing the Community Layer, the epidemic duration control rate and peak time control rate exhibit a non-linear change, first decreasing and then increasing, as the participation rate increases. This is likely because when the participation rate is low, the network becomes sparse, slowing the transmission process and extending the epidemic duration. When the participation rate is high, the spreading range significantly narrows, thereby shortening the epidemic duration. Under the strategy of migrating from the Academic Layer to the Community Layer, the duration control rate increases linearly with the participation rate, indicating that this strategy accelerates the epidemic’s spreading. The strategy of migrating from the Academic Layer to the Rest Layer shows a duration control rate that initially decreases and then increases, but the control rate remains negative throughout the observation period. This is because the Rest Layer has a smaller contact range than the Academic Layer, which slows the spread of the epidemic.

### 3.3. Impact of Zoned Management on Campus Epidemic Spreading

**Figure 5:**
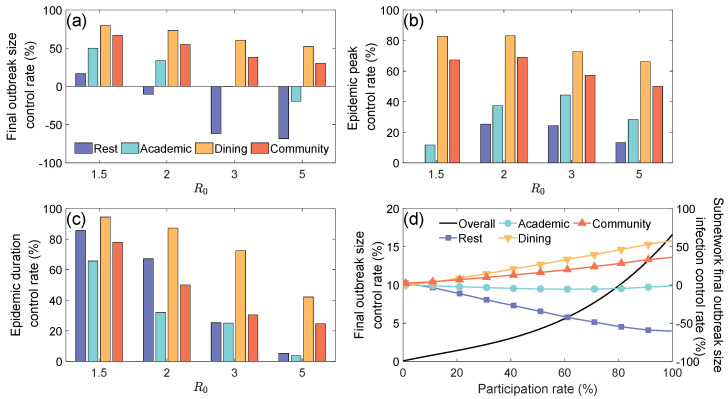
The impact of zoned management on epidemic control indicators and the changing effects of participation rates in zoned management on subnetworks. (**a**) Final outbreak size control rate (%) for different layers under varying $R_{0}$ values. Blue, cyan, yellow, and red represent the Rest, Academic, Dining, and Community layers, respectively. (**b**) Epidemic peak control rate (%) for each layer at different $R_{0}$ values. (**c**) Epidemic duration control rate (%) for each layer across varying $R_{0}$ values. (**d**) Final outbreak size control rate (%) as a function of participation rate, showing the overall infection control rate and infection control rate for each subnetwork.

Zoned management is an efficient epidemic prevention strategy at the university, which primarily involves dividing the student population into two roughly equal management areas, with non-overlapping contact zones. By physically isolating different functional areas while ensuring that each management area has complete facilities such as academic buildings and dormitories, this strategy can significantly reduce the contact density in high-mobility areas (e.g., Community and Dining Layers). However, since teaching and rest behaviors are typically confined to fixed regions, their contact patterns are less affected by zoned management. Figure 5 quantifies and evaluates the effectiveness of the zoned management through multiple dimensions. The three subgraphs (a)–(c) show the differentiated regulatory mechanisms of the zoned management on each subnetwork from the perspectives of epidemic peak control rate, the final outbreak size control rate, and epidemic duration control rate. Figure 5d illustrates how the overall final outbreak size control rate and subnetwork final outbreak size control rate vary with the participation rate of the zoned management measures.

Figure 5a shows that as the basic reproduction number $R_{0}$ increases, the final outbreak size control rate of all subnetworks decreases, with the Rest Layer showing the most significant decline. When $R_{0}$ is at a low level, the control effect of the Dining and Community Layers is better than that of the Rest and Academic Layers. When $R_{0}$ is high, the control rates for the Rest and Academic Layers become negative, meaning the infection numbers in these layers increase significantly. This phenomenon can be attributed to the dual effects of the zoned management strategy. On one hand, it effectively limits the interaction range of high-contact subnetworks (e.g., Community and Dining Layers), thereby inhibiting the spread of epidemics in these areas. On the other hand, the reduction in infection risk in the Dining and Community Layers causes the proportion of susceptible individuals in the Rest and Academic Layers to rise significantly compared with when the strategy is not implemented. With high $R_{0}$, the increased transmission efficiency leads to a sharp increase in the final outbreak size in the Rest and Academic Layers.

Figure 5b shows that as $R_{0}$ increases, the epidemic peak control rate for the Dining and Community Layers continues to decrease, while the Rest and Academic Layers show a non-monotonic change, first rising and then falling. This phenomenon is closely related to the cross-layer movement of susceptible individuals.

Figure 5c shows the change in epidemic duration control rates. As the basic reproduction number $R_{0}$ increases, the control effectiveness of the zoned management on epidemic duration gradually diminishes, with the most significant decline in the Rest and Academic Layers, while the Dining and Community Layers also show some degree of decline. This result suggests that as disease spreading ability strengthens, the suppressive effect of the zoned management on epidemic duration weakens, and the decay rate differs significantly between different functional areas.

As shown in Figure 5d, increasing the participation rate significantly enhances the epidemic controlling effect in the Dining and Community Layers, with no significant effect on the Academic Layer. However, the control rate in the Rest Layer decreases as the participation rate increases. This phenomenon can be explained from the following three perspectives: First, in areas with broader contact ranges (such as the Dining and Community Layers), the effect of zoned management measures is significantly enhanced as the participation rate rises, effectively reducing the final outbreak size in these areas. However, since the infection risk in the intervention areas is reduced, the proportion of susceptible individuals in the Rest and Academic Layers rises. Additionally, since the Rest Layer lacks targeted control measures, the final outbreak size in the Rest Layer increases, leading to a decline in the final outbreak size control rate. It is worth noting that although the Academic Layer shares similar crowd aggregation characteristics with the Rest Layer, due to restrictions on cross-area courses (e.g., elective courses across different majors), the transmission paths of infections are partially blocked, preventing significant changes in the final outbreak size control rate. This result suggests that prevention strategies must consider the dynamical coupling between subnetworks, avoiding localized optimizations that could cause systemic imbalances.

## 4. Discussion and Conclusions

We developed a multilayer temporal network model to rigorously examine the impact of student interactions on epidemic dynamics at the Minhang campus of East China Normal University. This model overcomes the limitations of traditional single-layer or temporal models by dividing campus activities into four subnetworks: the Rest Layer, Dining Layer, Community Layer, and Academic Layer. We focused on students’ time-varying migration behaviors between different locations and their contributions to epidemic spreading. The findings reveal that the Academic and Community Layers, with longer individual stay times and broader contact ranges, are the primary contributors to epidemic spread, while the Rest and Dining Layers pose relatively lower infection risks. By closing specific subnetworks and implementing zoned management strategies, epidemic spreading can be effectively controlled, though the effectiveness of these strategies is significantly influenced by the basic reproduction number $R_{0}$ and participation rates.

Notably, under low $R_{0}$ conditions, closing the Community Layer or implementing zoned management strategies can significantly reduce final size and epidemic peak, and shorten epidemic duration. However, as $R_{0}$ increases, the intervention effects on high-contact subnetworks (such as the Community and Dining Layers) become more pronounced, causing a relative increase in the susceptible population density in less-intervened subnetworks (such as the Academic and Rest Layers), which in turn heightens the infection risk in these areas. This result reveals a unique mechanism observable only with the use of multilayer temporal network structures—the “dynamic infection diversion effect”—highlighting the model’s innovation and superiority in capturing the dynamic characteristics of complex systems.

Based on these findings, we propose a tiered mitigation scheme:

1. Routine Control Phase ($R_{0}<2$): Focus on controlling the Community Layer, implementing reservation-based flow restrictions (≤200 people per time slot) and staggered time slots (strict control between 18:00 and 21:00);
2. Emergency Response Phase ($2\leq R_{0}<5$): Initiate partitioned management of the Academic Layer, splitting large classes (>100 people) into smaller sessions and restricting cross-area course selections;
3. Emergency State ($R_{0}\geq 5$): Implement grid-based management across the entire campus, creating independent living zones by dormitory buildings, along with online teaching and meal delivery measures.

Through this work, we provide theoretical foundations for epidemic prevention in universities, emphasizing the importance of considering the dynamic coupling between subnetworks in complex system modeling to avoid systemic imbalances due to local optimization. The proposed multilayer temporal network model uniquely captures transmission patterns beyond traditional methods, offering novel insights for epidemic control.

Future studies could incorporate faculty groups, individual behavior patterns, and social network structures to achieve more comprehensive characterization of campus transmission dynamics. For instance, examining how risk perception influences preventive measures among different campus populations (such as students, faculty, and staff) would provide deeper insights into transmission dynamics. This should include quantitative analysis of interlayer correlations and flow redistribution effects in the multiplex network framework, particularly how control measures in one layer (e.g., academic restrictions) propagate to others (e.g., community gatherings), with layer-specific transmission rates (β) reflecting distinct contact intensities (e.g., higher β in dense dining settings vs. lower β in outdoor community areas). The model could also benefit from considering how affinity-based interactions within social circles affect outbreak patterns, alongside existing factors like traffic flows and external inputs. Testing these refined approaches across diverse high-density environments—such as offices with hierarchical social structures or shopping centers with transient contacts—would further validate the model’s adaptability. Additionally, exploring variations in individual compliance with health protocols based on perceived risk could improve predictive accuracy for targeted interventions. Special attention should be given to the dynamic coupling between scheduled activities and emergent social network structures, where interlayer correlation strengths may evolve with policy changes or epidemic severity.

## Figures and Tables

**Table 1:** Typical daily class and routine schedule for students at East China Normal University (ECNU).

Time	Schedule
7:00–9:00	Breakfast
8:00–12:15	Class
11:00–13:00	Lunch
13:00–17:15	Class
17:00–19:00	Dinner
18:00–21:15	Class
22:30–6:30	Sleep

**Table 2:** Network model parameters and epidemic model parameters.

Parameter	Description	Value
*N*	Total population size	21,800
$N_{\mathrm{CO}}$	Number of colleges	29
$N_{M}$	Number of majors	58
$N_{G}$	Number of grades	4
$N_{\mathrm{Cou}}$	Total courses offered	2160
$C_{C}$	Class capacity	24–278
$C_{R}$	Dormitory capacity	2–4
$N_{D}$	Number of dining halls	10
$C_{D}$	Dining hall capacity	24–278
$R_{0}$	Basic reproduction number	1.5, 2, 3, 5
$\langle T_{E}\rangle$	Mean incubation period (days)	5.1 [31]
σ	Latent-to-infectious transition rate	$\sigma =1/T_{E}$
$\langle T_{I}\rangle$	Mean infectious period (days)	5 [32]
γ	Recovery rate	$\gamma =1/T_{I}$
β	Disease transmission rate	$\beta =R_{0}\times {\left(N\times T_{I}\right)}^{-1}$ [1]
$P_{R}$	Participation rate	0–100%
$T_{D}$	Time delay (days)	1, 2, 5, 7
$\langle k^{l}\rangle$	Average degree of subworks	$\langle k^{D}\rangle =3.11$, $\langle k^{C}\rangle =7.94$,
		$\langle k^{R}\rangle =3.06$, $\langle k^{A}\rangle =3.89$ [33]

## Data Availability

The data used in this study are available from the corresponding author upon reasonable request.
